# Hospital Administration

**Published:** 1895-07-20

**Authors:** 


### HOSPITAL ADMINISTRATION.

### The Lines System.

There is one thing connected with the Scotch hospitals, and especially with the Glasgow hospitals, which creates a great deal of unnecessary friction and makes it very difficult at times to secure that the hospital shall husband its resources by making them available only for such cases as really need the assistance they can supply. The lines system practically represents the worst features of the ticket system, now, happily, almost a dead letter in England. The subscriber gives so many pounds per annum and receives in exchange in-patient and out-patient lines. The foreman of one of the subscriber's departments falls ill, I will suppose. He is a man in good circumstances, with a comfortable home, and his ailment is trivial in character. The master, however, finding that the foreman will be absent from work for a time, gives him one of his in-patient lines, and sends him off to the hospital, although he may not be, and probably is not, in any proper meaning of that term, a suitable patient for treatment. If the medical officer sees the case, and declines to admit it as a medical case owing to its trivial character, then the subscriber writes an indignant letter to the hospital authorities, and sends it back with the patient to the institution. If the patient is again rejected, it frequently happens that the employer writes to say that as his lines are no good to him, he shall discontinue his subscription. In practice, the employer treats his subscription and his foreman's illness from the financial aspect only. The lines only cost him a certain sum, and it is not his business what the treatment of the in-patient may cost the hospital. If the foreman remains at home ill, there are his wife and children to be considered, and he cannot in decency reduce, much less withhold, his wages. If the man is received into the hospital, however, in virtue of the subscriber's lines, then the employer, being a man of that ilk, has an opportunity of doing what he may regard as the true basis of business, thereby saving his pocket. The man may be kept by the hospital a fortnight, and as he is in the hospital there will be no expenditure at home on his behalf, so, as he is not working, the employer feels justified in reducing or withholding his wages. The sum thus saved to the employer at the expense of the hospital was, we were informed, much larger than the subscription given by the employer to the hospital. We think that the hospitals in Glasgow and elsewhere will be well advised to take this question boldly in hand, to extend its ramifications in both cases, and then to make an appeal to their subscribers to aid the managers in protecting the institutions from an abuse, and the medical staff from worries, they never ought to have to face. At any rate, this lines system is well worthy a free discussion, and we hope our calling attention to it may, at any rate, attain this end.

